# A novel deep intronic variant in *ATP7B* in five unrelated families affected by Wilson disease

**DOI:** 10.1002/mgg3.1428

**Published:** 2020-08-08

**Authors:** France Woimant, Aurelia Poujois, Adrien Bloch, Tabaras Jordi, Jean‐Louis Laplanche, Hélène Morel, Corinne Collet

**Affiliations:** ^1^ National reference Centre for Wilson’s Disease (CRMR Wilson) Department of Neurology Rothschild Hospital Paris France; ^2^ Department of Neurology Lariboisiere University Hospital APHP Paris France; ^3^ Department of Biochemistry and Molecular Biology Lariboisiere University Hospital APHP Paris France; ^4^ INSERM U1132 University Paris‐Diderot and Department of Rheumatology Lariboisiere University Hospital Paris France

**Keywords:** *ATP7B*, deep intronic variant, splicing, Wilson disease

## Abstract

**Background:**

Wilson disease is an autosomal recessive metabolic disorder resulting from accumulation of excess copper especially in the liver and brain. This disease is mainly characterized by hepatic disorders and less frequently by neuro‐psychiatric disturbances. This recessive disease is due to mutation in *ATP7B*, which codes for an ATPase involved in copper‐transport across the plasma membrane. Molecular diagnosis of WD is positive in approximately 98% of cases. Also, in few cases, WD patients present a single deleterious mutation (heterozygous) or no mutation after sanger and NGS standard sequencing analysis of *ATP7B*. Therefore, in these problematic WD cases, we hypothesized that deleterious mutations reside in intronic regions of *ATP7B*.

**Methods:**

Complete *ATP7B* gene was sequenced by Next Generation Sequencing including its promoter.

**Results:**

Five unrelated families with Wilson disease shared the same novel, deep intronic NG_008806.1 (ATP7B_v001):c.2866‐1521G>A variant in *ATP7B*. Analysis of RNA transcripts from primary fibroblasts of one patient confirmed the deleterious impact of the intronic variant on splicing and its likely pathologic effect in this compound heterozygote.

**Conclusion:**

This discovery of a novel intronic mutation in *ATP7B* has improved the molecular diagnosis of WD in the French patient cohort to greater than 98%. Thus, we recommend complete sequencing of *ATP7B* gene, including introns, as a molecular diagnostic approach in cases of clinically confirmed WD which lack pathogenic exon or promoter variants in one or both alleles.

## Graphical abstract

Unexpected intronic splicing variant in Wilson disease.

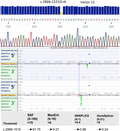

## 1 INTRODUCTION

Wilson disease (WD) is a rare autosomal recessive monogenic disorder due to excess copper deposition, affecting especially the brain and liver. Mutations in the *ATP7B* gene (OMIM 606882), which codes for an ATPase involved in copper‐transport across the plasma membrane, are responsible for this pathology. This ATPase incorporates copper into apo‐ceruloplasmin which is released into the serum. WD results from defective biliary excretion of copper that leads to its accumulation (Polishchuk & Polishchuk, 2019). The onset of symptoms is variable, mainly characterized by hepatic disorders and neuro‐psychiatric disturbances (Pfeiffer, 2016). WD mostly appears in children and young adults; however, it may present at any age between 3 and 80 years old (Bandmann, Weiss, & Kaler, 2015). The hepatic form ranges from asymptomatic with only biochemical abnormalities to cirrhosis, acute hepatitis, and chronic hepatitis. Neurologic or psychiatric signs may be the first clinical manifestation, or they may appear simultaneously with hepatic signs or some years later. The neurologic form is characterized by tremor, dysarthria, parkinsonism, ataxia, and dystonia, while the psychiatric signs vary according to the age of onset, ranging from inappropriate behavior to schizophrenia or depression (Poujois, Mikol, & Woimant, 2017). Lifelong drug treatments or hepatic transplant in the case of fulminant hepatitis or decompensated cirrhosis allow a normal life in the majority of WD patients.

At the population level, WD is characterized by a variable and low clinical prevalence, ranging from 1 to 9 per 100,000 depending on the population (Poujois et al., 2018). However, the frequency of heterozygous carriers is higher than expected in France (1/31), the United Kingdom (1/25), and Korea (1/55) based on the respective clinical prevalence in those countries (Coffey et al., 2013; Collet, Laplanche, et al., 2018; Collet, Woimant, Laplanche, & Poujois, 2018; Jang, Lee, Bang, Kim, & Cho, 2017). This observed difference between the clinical and molecular prevalence is currently unexplained, but misdiagnosis, under‐diagnosis, modifier genes, and environmental factors are the main hypotheses.

Molecular diagnosis of WD, which is based on sequencing the coding region of *ATP7B* and its promoter, is positive in approximately 98% of cases (Collet, Woimant, et al., 2018). However, the remaining 2% of WD patients, with a classic hepatic or neurologic phenotype and abnormal copper balance, show only a single deleterious mutation (heterozygous) or no mutation after standard sequencing analysis of *ATP7B*. Therefore, in these problematic WD cases, we hypothesized that deleterious mutations reside in intronic regions of *ATP7B*. Here, we tested this hypothesis by sequencing the entire *ATP7B* gene, including introns, from 10 such WD families.

## 2 MATERIALS AND METHODS

### 2.1 Patients

From a total of 442 index cases diagnosed with WD, we selected those patients having only a single mutation or no mutation detected in the *ATP7B* gene. All selected WD patients provided from the cohort of Lariboisière Hospital in Paris. Before implementation of NGS technology in our laboratory in 2015, all the selected patients from the cohort were screened for *ATP7B* mutations by Sanger sequencing and multiplex ligation‐dependent probe amplification (MLPA) technology at diagnosis. Informed consent was obtained for all patients or their families and the study was approved by a French ethics committee. Clinical characteristics, imagery diagnosis, and abnormal copper levels were reviewed for all WD patients. All included patients had a Ferenci score >4 and were treated. WD diagnosis was confirmed in all WD patients. All tested controls were free of WD.

### 2.2 Next Generation Sequencing for whole ATP7B gene study

DNA samples were screened by NGS using a design including the entire 75‐kb sequence of *ATP7B*. We used the following nomenclature: *ATP7B* (NM_000053.4) for the exonic regions and intronic‐exonic boundaries; *ATP7B* (NG_008806.1) for exon numbering and the intronic regions.

Libraries for NGS were established using the SureSelectXT kit (Agilent, Les Ulis, France) based on a hybrid capture system for sequencing on the Miseq sequencer (Illumina, Paris, France). The bioinformatics pipeline included the miseq‐reporter (Illumina) to generate Fastq files. This was followed by Fastq alignments with SeqNext and visual variant control (JSI Medical Systems, Ettenheim, Germany) and Alamut (https://www.interactive‐biosoftware.com). The variants were included if the depth of coverage was greater than 30‐fold, with a heterozygous variant frequency ranging from 0.30 to 0.60 and homozygous variant frequency ranging from 0.6 to 1. Studies of variant pathology involved the use of predictive software programs such as PhyloP (http://compgen.cshl.edu/phast/), Polyphen‐2 (http://genetics.bwh.harvard.edu/pph2/), CADD (https://cadd.gs.washington.edu), and MutationTaster (http://www.mutationtaster.org), whereas splicing variant pathology was determined using predictive software such as GeneSplicer (http://www.cbcb.umd.edu/software/GeneSplicer), MaxEntScan (http://genes.mit.edu/burgelab/maxent), NNSPLICE (http://www.fruitfly.org/seq_tools/splice), and SSF (www.umd.be/searchSpliceSite).

The frequencies of all variants were searched in GnomAD (gnomad.broadinstitute.org/), Exome Sequencing Project (ESP, evs.gs.washington.edu), and dbSNP (https://www.ncbi.nlm.nih.gov/projects/SNP).

### 2.3 Sanger sequencing

After amplification with herculase II (Agilent, Les Ulis, France), Sanger sequencing was performed with Thermo fisher reagents according to the manufacturer's protocol on an ABI3130 sequencer (Thermo fisher, Saint Herblain, France) and then, analyzed with seqscape v4.0 software (Thermo fisher).

### 2.4 Functional study

Primary skin fibroblast cultures were obtained using explants from skin punch biopsies from one patient and two controls. The skin biopsy of the patient was performed after informed consent.

Cells were grown in high‐glucose Dulbecco's modified Eagle's medium (DMEM) supplemented with 10% (v/v) fetal bovine serum (FBS), 1% (v/v) L‐glutamine, and 1% (v/v) penicillin/streptomycin, at 37°C in a humidified atmosphere of 5% CO_2_. All cell cultures were established with reagents from Thermo fisher. Cells from each biological repeat (i.e., from three independent fibroblast cultures) were used for RNA sample preparation.

### 2.5 RNA isolation, cDNA synthesis, and quantitative RT‐PCR

RNA was isolated using an RNA kit (Qiagen, Couraboeuf, France). After DNase treatment, cDNA was synthesized using SuperScript III Reverse Transcriptase (Thermo fisher).

PCRs were performed using Qiagen reagents and two pairs of primers designed to anneal to exon 7 (5′GACCTGCGCGTCCTGTGTCCA3′) and exon 13 (5′CTTGTGCGCCATCTCCAGGGG3′) or exon 5 (5′GAAACCACAGTGCTGGGAATT3′) and exon 14 (TACAGTATTTGGTGACTGCCC).

PCRs were analyzed in a 1% agarose gel and visualized with a gel‐imaging system (BioRad, Les Ulis, France). The PCR products were purified with a NucleoSpin® Gel kit (Macherey‐Nagel, Hœrdt, France) according to the manufacturer's instructions and then, sequenced with different pairs of primers.

## 3 RESULTS

### 3.1 Frequency of WD patients with no or one mutation detected in *ATP7B*

Among the 442 WD index cases in the Lariboisière cohort, we found eight patients with one mutation in *ATP7B* and two patients with no mutation. The clinical characteristics, treatments, and copper levels of the 10 patients were presented in Table 1. All patients were diagnosed as WD. No consanguinity was noted for these 10 index cases and all were unrelated (Table 2).

**Table 1:** Clinical characteristics and treatment of 10 WD patients

Case	Sex	Year of diagnosis	Age at diagnosis (years)	Phenotype at diagnosis	First symptoms	KFR	Leipzig score	Initial treatment
1	M	2014	23	Hepatic	Fulminant hepatitis with hemolytic anemia	No	6	Liver transplant
2	M	1986	11	Hepatic	Liver cytolysis and cirrhosis	Yes	7	D Penicillamine
3	F	2005	23	Neurohepatic	Tremor	Yes	9	D Penicillamine
4	F	1998	32	Neurohepatic	Liver failure and tremor	Yes	8	D Penicillamine
5	M	1981	7	Hepatic	Liver cytolysis	No	7	D Penicillamine
6	M	2012	22	Hepatic	Liver cytolysis	No	5	Zinc acetate
7	F	2012	24	Neurohepatic	Tremor, depression, gait troubles	Yes	7	D Penicillamine
8	M	2016	42	Hepatic	Liver cytolysis, improved under chelator	No	4	Trientine
9	F	2006	19	Hepatic	Cirrhosis with hemolytic anemia	No	5	D Penicillamine
10	F	1995	31	Hepatic	Liver cytolysis	No	4	D Penicillamine

Note

KF = Kayser‐Fleischer ring. Leipzig score is usually used in WD (Roberts & Schilsky, 2003).

**Table 2:** Copper levels and molecular results of 10 WD patients

Case	CP	Serum copper	Urinary copper	REC	Hepatic copper values	Pathogenic mutations
	*N* > 0.20 g/L	*N* > 12;7 µmol/L	*N* < 0.40 µmol/L	*N* < 8%	*N* < 4 µmol/g of dry weight liver tissue	
1	<0.01	7.98	no data	18.9	8.5	NG_008806.1(ATP7B_v001): c.2866‐1521G>A NM_000053.4: c. 3551T>C, p.(Ile1184 Thr)
2	0.04	No data	4	No data	No data	NG_008806.1(ATP7B_v001): c.2866‐1521G>A NM_000053.4: c.3207C>A, p.(His1069Gln)
3	0.05	6	4.95	No data	No data	NG_008806.1(ATP7B_v001): c.2866‐1521G>A NM_000053.4: c.3121C>T, p.(Arg1041Trp)
4	0.09	No data	1.2	No data	No data	NG_008806.1(ATP7B_v001): c.2866‐1521G>A NM_000053.4: c.3207C>A, p.(His1069Gln)
5	0.02	5.3	2.8	No data	21.6	NG_008806.1(ATP7B_v001): NM_000053.4: c.2866‐1521G>A NM_000053.4: c.3182G>A
6	0.08	3.85	1.14	31.2	No data	NM_000053.4: c.122A>G p.(Asn41Ser)
7	0.09	8.89	2.95	30.7	No data	NM_000053.4: c.3207C>A p.(His1069Gln)
8	0.11	6.11	0.73	12	2.1	NM_000053.4: c.3083_3085delinsG, p.(Lys1028Serfs*40)
9	N (but hemolysis)	N (but hemolysis)	3.1	No data	15.7	No mutation
10	0.14	4.19	1.88	No data	4.9	No mutation

Note

CP = Ceruleoplasmin level, Cu hep (µmol/g), REC, Relative exchangeable copper.

To complete the molecular analysis and confirm the original Sanger sequencing data for each of these patients, we examined the promoter region, the coding region, and the 5′ and 3′UTR by NGS and looked for copy number variants (CNV) by MLPA and NGS. These analyses failed to find any novel variant or unreported deleterious variant that could explain the disease. Therefore, the frequency of non‐informative molecular results in this cohort is 2.3% (10/442).

### 3.2 Sequencing the entire *ATP7B* gene by NGS

For the 10 WD index cases with only one mutation (n = 8) or no mutation (n = 2) detected initially, we sequenced the entire *ATP7B* gene, a total of 75 kb. We achieved ~93% coverage at ≥30‐fold, with an average coverage of 250‐fold. A few intronic regions with repeat nucleotides were not sequenced. Otherwise, gaps in the sequence were filled using the Sanger technique, except for a 5‐kb segment corresponding to the genomic region g.52522135C>T to g.52527135C>T. In the eight patients with a heterozygous mutation in an exon or exon‐intron junction previously found by Sanger sequencing, we confirmed the presence of those mutations.

In five patients, three with a single mutation and the two patients with no mutation, no additional mutations were identified after sequencing the entire *ATP7B* gene.

**Figure 1:**
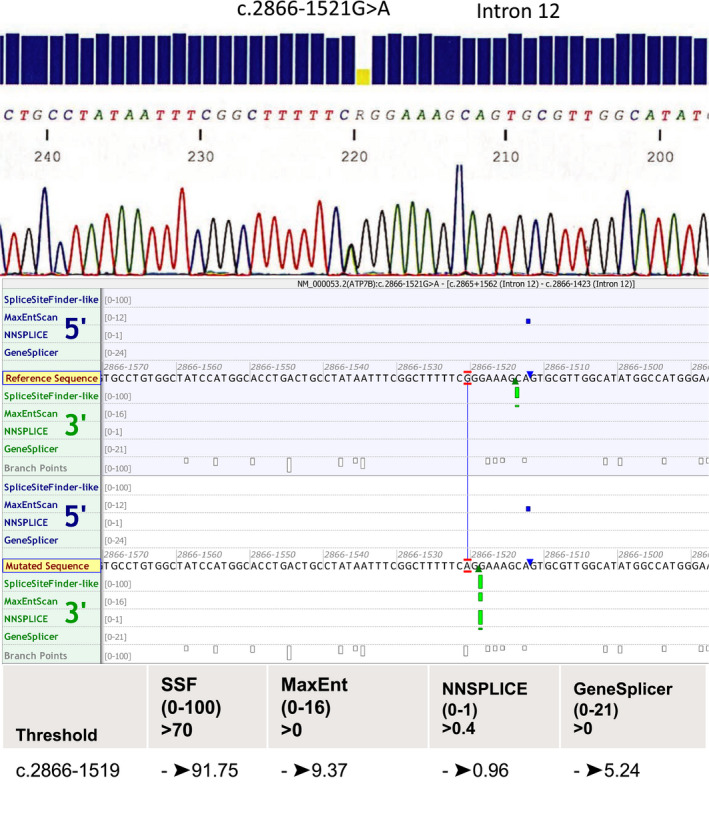
Electropherogram of Sanger sequencing results of the intronic variant NG_008806.1 (ATP7B_v001):c.2866‐1521G>A in *ATP7B* (the mutated site is heterozygous). All predictive software showed creation of an acceptor splicing site in this variant

In all five of the remaining patients, we detected the same novel intronic variant, NG_008806.1 (ATP7B_v001):c.2866‐1521G>A, as a heterozygous mutation. This variant in intron 12 affects a conserved position according to PhyloP and is not listed in GnomAD and dbSNP. According to splicing predictor software (SSF, MaxEnt, GeneSplicer, NNSplice), this intronic variant would create an acceptor site in the middle of intron 12, with high scores (Figure 1). We confirmed the presence of this intronic variant in all five patients by Sanger sequencing (Figure 1). None of 100 control subjects tested by dHPLC carried this intronic variant. The presence of the intronic NG_008806.1 (ATP7B_v001):c.2866‐1521G>A variant in five patients with WD corresponds to 1.13% of our WD cohort. Taken together, these results support a deleterious effect of the variant on *ATP7B*.

### 3.3 Clinical phenotype and familial segregation

The clinical and biological characteristics of the five index patients who share the intronic variant are presented in Table 1. These WD patients were diagnosed at different ages and displayed variable onsets and good evolution. No specific phenotype seems to be associated with this intronic variant.

**Figure 2:**
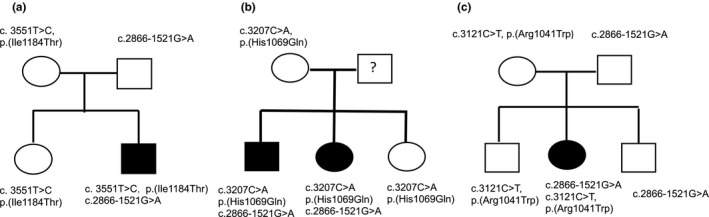
Family trees of patients with WD who carry the intronic variant NG_008806.1(ATP7B_v001):c.2866‐1521G>A. (a) Family 1: The index case is a compound heterozygote for the missense mutation NM_000053.4:c.3551T>C, p.(Ile1184 Thr), and the intronic variant NG_008806.1(ATP7B_v001):c.2866‐1521G>A. (b) Family 2: Both affected patients are compound heterozygous for the missense mutation NM_000053.4: c.3207C>A, p.(His1069Gln), and the intronic variant NG_008806.1(ATP7B_v001):c.2866‐1521G>A. The father of the affected patients was not available and his genotype is indicated as “?”. (c) Family 3: The index case is a compound heterozygote for the missense mutation NM_000053.4: c.3121C>T, p.(Arg1041Trp), and the intronic variant NG_008806.1(ATP7B_v001):c.2866‐1521G>A

For three of the five affected families, we were able to determine the *ATP7B* genotype of other family members in addition to the index case (Figure 2). For family 1, the index case carries the NM_000053.4: c.3551T>C, p.(Ile1184Thr) variant in exon 16 (Bost, Piguet‐Lacroix, Parant, & Wilson, 2012) and the NG_008806.1(ATP7B_v001):c.2866‐1521G>A intronic variant. In family 2, both individuals affected by WD carry the mutation NM_000053.4: c.3207C>A, p.His1069Gln in exon 14 along with the intronic variant. The sister of the index case was pauci‐symptomatic at screening and was started on D Penicillamine treatment. In family 3, the index case carries the mutation NM_000053.4:c.3121C>T, p.(Arg1041Trp) in exon 14 and NG_008806.1(ATP7B_v001):c.2866‐1521G>A.

For the two other index cases, the novel intronic variant was associated with the NM_000053.4: c.3207C>A p.His1069Gln variant in index case 4 and with the NM_000053.4:c.3182G>A p.Gly1061Glu variant (Curtis et al., 1999) in index case 5 (familial studies were not performed because their DNAs were not available).

Therefore, the finding that compound heterozygotes carrying the NG_008806.1(ATP7B_v001):c.2866‐1521G>A variant in intron 12 together with any one of four different missense mutations in *ATP7B* underscores the deleterious nature of the intronic variant and its contribution to WD. However, it was important to confirm the molecular defect by functional studies.

### 3.4 Functional study of the intronic variant in *ATP7B*

To confirm the deleterious effect of the NG_008806.1(ATP7B_v001):c.2866‐1521G>A intronic variant on *ATP7B* transcript production, we prepared primary skin fibroblast cultures from skin biopsies from case 1 and two unrelated controls.

**Figure 3:**
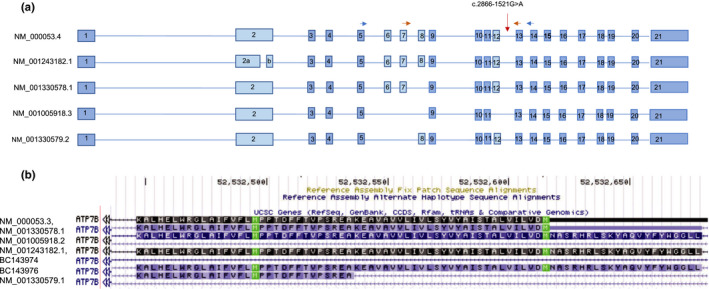
(a) The five major transcripts of the *ATP7B* gene with or without the central exons (6, 7, 8). The horizontal arrows designate the primer pair used to amplify exons 7–13 (red) or exons 5–14 (blue). (b) Graphic of the reference transcripts of *ATP7B* in UCSC focused on exon 8. BC143976 is a transcript with a smaller exon 8; the other transcripts include or do not include the entire exon 8

From cDNA, PCRs were performed with two different pairs of primers to examine the effect of the intronic variant on the *ATP7B* transcript profile. One primer pair was designed to amplify from exon 5 to 14 and the other primer pair to amplify from exon 7 to 13 in order to frame intron 12 (Figure 3).

**Figure 4:**
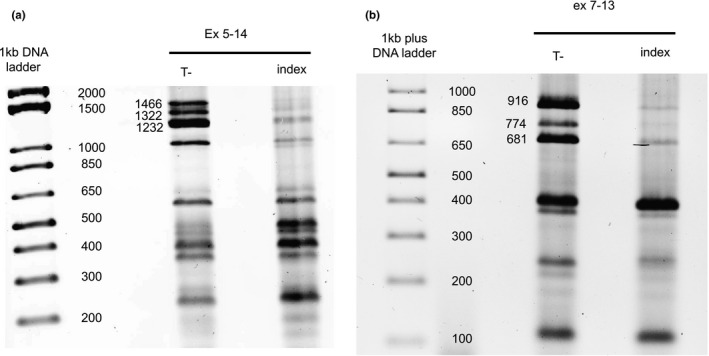
Comparison of the profiles of *ATP7B* transcripts between one control and a patient who carries the NG_008806.1(ATP7B_v001):c.2866‐1521G>A intronic variant. (a) Transcripts detected with the primers in exon 5 and exon 14; the longest transcript (NM_000053.4 = 1466 bp) was nearly completely absent in the patient with the intronic variant. (b) With the primer pair designed to amplify exon 7 to 13, the longest transcript (NM_000053.4 = 916 bp) was nearly completely absent in the patient with the intronic variant. This experiment was repeated in triplicate and a representative image is shown

As shown in Figure 4, the *ATP7B* transcript profile of the patient is markedly different from that of the control for both primer pairs. Especially notable is the underrepresentation of the longest transcripts in the patient compared to the control.

The *ATP7B* gene has five transcripts referenced in RefSeq NCBI (NM_000053.4, NM_001243182.1, NM_001005918.2, NM_001330578.1, and NM_001330579.1), which differ in their central exons (Figure 3). The longest transcript is represented by NM_000053.4 and includes 21 exons. Exons 1 to 5, 9, 10, 11, 13 to 21 are common to four transcripts (NM_001005918.2, NM_001330578.1, NM_001330579.1, and NM_000053.3). In the smallest transcript (NM_001005918.2), exons 6, 7, 8, and 12 are absent, and in the NM_001243182.1 transcript, exon 2 is divided in two. Also, transcript NM_001330578.1 has no exon 6 and 7 and transcript NM_001330579.1 has no exon 8. Additionally, among the various predicted transcripts (mRNA from the GenBank database), there is one with a smaller exon 8 (90 bp) corresponding to BC143976 (Figure 3).

For the control samples, the longest transcript was detected with the primers designed to anneal to exons 5 and 14 (1466 bp) and also with the primers designed to anneal to exons 7 and 13 (915 bp). Sanger sequencing of the control PCR products amplified using the primers in exons 7 and 13 confirmed the presence of the longest transcript, as did sequencing of the PCR products generated with the primers in exons 5 and 14.

By agarose gel electrophoresis, the control PCR products displayed an expected size of 1232 bp using the primers in exons 5 and 14, and of 680 bp with the primers in exons 7 and 13, corresponding to the transcript without exon 8 (234 bp) (variant NM_001330579.1). Moreover, the presence of the transcript was confirmed by Sanger sequencing with both primer pairs. The PCR product (1322 bp) intermediate in size between the two previously described transcripts could correspond to the predicted BC143976 transcript with a short (90 bp) exon 8 (Figure 3).

In summary, the levels of the three long transcripts were substantially reduced in the presence of the NG_008806.1 (ATP7B_v001):c.2866‐1521G>A intronic variant as compared to controls. In contrast, the shortest transcripts were present irrespective of the mutation. These results confirm that the NG_008806.1 (ATP7B_v001):c.2866‐1521G>A intronic variant causes a significant reduction in long *ATP7B* transcripts, thereby resulting in a nonfunctional transporter in the presence of a second deleterious allelic mutation.

## 4 DISCUSSION

Our report describes for the first time a deleterious *ATP7B* mutation, NG_008806.1 (ATP7B_v001):c.2866‐1521G>A, deep within an intron (intron 12) in five index patients with WD, thus present in 1.13% of patients in our cohort. This intronic variant disrupts transcript processing, especially of the long transcripts. Absence of the ATP7B transporter has largely explained WD pathology. However, so far, no phenotype–genotype correlations have been described for the other referenced deleterious variants in WD (Chang & Hahn, 2017).

Our work shows the importance of sequencing the whole ATP7B gene in cases of exon sequencing is non‐informative. ATP7B genome sequencing could be used to extend the molecular analysis. Importantly, patients in five unrelated families were found to share the same deleterious intronic mutation NG_008806.1 (ATP7B_v001):c.2866‐1521G>A. These patients all came from different parts of France. So, it would be interesting to generalize our finding to other populations. Genome sequencing could be used to extend the molecular analysis of those patients for whom only a single pathologic mutation has been detected. Genome sequencing was also recently used by Chen et al. (2018) to identify a pathologic homozygous variant in the promotor region of *ATP7B*.

In addition, our approach allowed us to resolve half (5/10) of the undefined molecular cases in our WD cohort. In turn, we were able to define the genotypes of related persons in three cases of familial screening. The molecular screening that we applied included sequencing of the promotor and the 5′ and 3′ UTR regions of *ATP7B*. The fact that we were not able to detect a deleterious mutation in five other patients carrying no or only one detected *ATP7B* mutation could indicate that the unidentified mutations reside in a regulatory region close to *ATP7B*; for example, a large heterozygous deletion would be undetectable by our approach. Another possibility is misdiagnosis of WD, which occurs in about 1% of cases, as the list of differential diagnoses of WD is long and includes manganese storage disease with symptoms similar to those of WD (Hermann, 2019).

The primary fibroblast cell culture model that we used to examine splicing was able to confirm the deleterious nature of the splicing variant NG_008806.1 (ATP7B_v001):c.2866‐1521G>A. Our initial tests using RNA extracted from lymphocytes collected in paxgene tubes showed the same results but the transcription of *ATP7B* was very low in both control and mutated cells, rendering the sequencing analyses difficult (data not shown). Interestingly, the five different *ATP7B* transcripts listed in RefSeq NCBI differ in the central exons (exons 6, 7, 8, 9), and exon 8 is especially mutated in WD (Wang et.al., 2018), suggesting a critical function for this region of the ATP7B protein. However, various predicted transcripts in different databases and the results of our own study also indicate the probable existence of a transcript with a short exon 8, detectable in fibroblasts (transcript BC143976 in Figure 3). Expression profiling in different tissues and/or RNAseq analyses in single cells will be necessary to begin to understand the roles of the different predicted *ATP7B* transcripts and the protein variants they may produce.

In conclusion, we described a novel, deep intronic NG_008806.1(ATP7B_v001):c.2866‐1521G>A variant in *ATP7B* segregating in five unrelated families with WD. Analysis of RNA transcripts from primary fibroblasts of one patient confirmed the deleterious nature of the intronic variant on splicing and, therefore, its likely pathologic effect in this compound heterozygote. Our discovery of a novel intronic mutation in *ATP7B* has improved the molecular diagnosis of WD in the French patient cohort to greater than 98%. Thus, we recommend complete sequencing of *ATP7B* as a molecular diagnostic approach in cases of clinically confirmed WD in which pathogenic exon or promoter variants in one or both alleles have not been detected.

## CONFLICT OF INTEREST

No declaration.

## AUTHOR'S CONTRIBUTION

C.C, F.W and A.P designed the work. F.W and A.P. provided the clinical and biological information of the patients. A.B., J. T., and H. M. carried out the experiments and molecular genetic analysis. C.C. wrote the manuscript with support from J.L.L, A.P., and F.W. C.C conceived the original idea. All authors approved the final version.
